# A-Kinase Interacting Protein 1 Promotes Cell Invasion and Stemness *via* Activating HIF-1α and β-Catenin Signaling Pathways in Gastric Cancer Under Hypoxia Condition

**DOI:** 10.3389/fonc.2021.798557

**Published:** 2022-03-09

**Authors:** Zhenqin Luo, Yuhang Luo, Ke Xiao

**Affiliations:** ^1^ Department of Comprehensive Chemotherapy, Hunan Cancer Hospital and The Affiliated Cancer Hospital of Xiangya School of Medicine, Central South University, Changsha, China; ^2^ Department of Hepatobiliary Surgery, The Second Affiliated Hospital, The University of South China, Hengyang, China; ^3^ Department of Gastroduodenal and Pancreatic Surgery, Hunan Cancer Hospital and The Affiliated Cancer Hospital of Xiangya School of Medicine, Central South University, Changsha, China

**Keywords:** gastric cancer, AKIP1, invasion, stemness, HIF-1α and β-catenin pathways

## Abstract

**Background:**

A-Kinase interacting protein 1 (AKIP1) relates to gastric cancer growth, metastasis, and prognosis, while its regulation on gastric cancer invasion and stemness under hypoxia microenvironment is not reported. Therefore, this study aimed to explore this topic to uncover AKIP1’s role in gastric cancer under hypoxia.

**Methods:**

Gastric cancer cell lines AGS and MKN45 were cultured under hypoxia condition, then transfected with AKIP1 or negative control (NC) overexpression plasmid or AKIP1 or NC knockdown plasmid. Furthermore, rescue experiments were conducted by transfecting HIF-1α or β-catenin overexpression plasmid, combined with AKIP1 or NC knockdown plasmid. Afterward, cell invasion, CD133^+^ cell proportion, sphere number/1,000 cells, and HIF-1α and β-catenin pathways were measured.

**Results:**

The invasive cell count, CD133^+^ cell proportion, and sphere number/1,000 cells were enhanced in both AGS cells and MKN45 cells under hypoxia, and AKIP1 expression was also elevated. AKIP1 knockdown inhibited cell invasion, CD133^+^ cell proportion, sphere number/1,000 cells, HIF-1α, vascular endothelial growth factor (VEGF), β-catenin, and calcium-binding protein (CBP) expressions in AGS cells and MKN45 cells under hypoxia, while AKIP1 overexpression presented with the opposite effect. Then, in rescue experiments, HIF-1α overexpression and β-catenin overexpression both promoted cell invasion, CD133^+^ cell proportion, and sphere number/1,000 cells, which also attenuated the effect of AKIP1 knockdown on these functions in AGS cells and MKN45 cells.

**Conclusion:**

AKIP1 promotes cell invasion and stemness *via* activating HIF-1α and β-catenin signaling pathways in gastric cancer under hypoxia condition.

## Introduction

Gastric cancer remains to be one of the most frequent and fatal cancers in the world, despite the reduction in risk factors such as *Helicobacter pylori* (1–4). With the aging of the population, the incidence of gastric cancer will be increasing, and what is worse is that the early disease is hard to be discovered under usual examination; most patients are diagnosed with advanced disease (5, 6). Gastric cancer treatment demands a multidisciplinary work; luckily, insights of the pathology of gastric cancer keeps growing, which has largely contributed to the exploration of targeted therapy, immune therapy, or updated chemotherapy regimen (7–9). Hypoxia, an imperative feature in cancer development, is both a cause and a consequence in tumor progression, and in recent decades, hypoxia as a treatment target is increasingly indicated in cancer treatment (10–12).

A-Kinase interacting protein 1 (AKIP1), a small protein of 23 kDa, regulates the signaling of cAMP-dependent protein kinase, which belongs to the cascade of NF-kappa-B activation (13, 14). Most importantly, AKIP1 is also a promising oncology-related factor in recent studies. For instance, AKIP1 is upregulated in several cancers, and it also correlates with more deteriorative tumor features and survival profiles in these cancer patients, which suggests that it participates in the progression of many cancers (15–19). Above all, there is also correlation between AKIP1 and gastric cancer, which elucidates that it is involved in tumor progression through the modulation of Slug-induced epithelial–mesenchymal transition, which then enhances growth and metastasis of the tumor (20). Therefore, AKIP1 may also be involved in the regulation of gastric cancer pathogenesis under hypoxia, while there is no evidence supporting this presumption.

Hence, the present study aimed to investigate the effect of AKIP1 on cell invasion and stemness in gastric cancer under hypoxia, and its interaction with hypoxia-inducible factor 1 subunit alpha (HIF-1α) and β-catenin pathways.

## Methods

### Cell Culture and Hypoxia Treatment

GC cell lines AGS and MKN45 were purchased from China Center for Type Culture Collection (CCTCC, China) and Japanese Collection of Research Bioresources (JCRB) Cell Bank (JCRB, Japan), separately. The 90% Ham’s F-12K medium (GIBCO, USA) with 10% fetal bovine serum (GIBCO, USA) was used for the culture of AGS cells. The 90% Roswell Park Memorial Institute (RPMI) 1640 medium (GIBCO, USA) with 10% fetal bovine serum (GIBCO, USA) was used for the culture of MKN45 cells. The cell culture condition in normal oxygen was at 37°C in a humidified atmosphere of 5% CO_2_ and 21% O_2_, while for hypoxia treatment, the cell culture was performed in a hypoxia cell incubator of 5% CO_2_ and 0.5% O_2_ at 37°C. To assess the influence of hypoxia on the GC cells, we first divided AGS and MKN45 cells into two groups: (a) control group, which was cultured in the normal oxygen condition, and (b) hypoxia group, which was cultured in the hypoxia condition. After 48-h culture, the Transwell assay, flowcytometry, sphere formation assay, reverse transcription quantitative polymerase chain reaction (RT-qPCR), and Western blot were performed.

### Plasmid Construction and Transfection

The pEX-2 vector (Genepharma, China) was applied as a carrier for construction of AKIP1 overexpression [AKIP1(+)] plasmid and negative control (NC) overexpression [NC(+)] plasmid; the pGPH1 vector (Genepharma, China) was used as a carrier for construction of AKIP1 knockdown [AKIP1 (–)] plasmid and NC knockdown [NC(−) plasmid. The constructed plasmids were respectively transfected into AGS cells and MKN45 cells using Lipofectamine™ 3000 Transfection Reagent (Invitrogen, USA) following the instructions of the manufacturer, and the resulting cells were respectively named as AKIP1(+), NC(+), AKIP1(−), and NC(−) in each cell line. The cells without transfection were used as normal control. After transfection, all cells were incubated for 48 h under the hypoxia condition as described in the “*Cell Culture and Hypoxia Treatment*”. The Transwell assay, flow cytometry, sphere formation assay, RT-qPCR, and Western blot were performed.

### HIF-1α and β-Catenin Inhibition

YC-1 and LF3, the inhibitors of HIF-1α and β-catenin pathways, were purchased from MedChemExpress (MCE, China). The concentration of YC-1 or LF3 incubating with cells in was 10 or 30 μM. After incubated under hypoxia condition for 48 h, the Transwell assay, flow cytometry, and sphere formation assay were performed.

### Rescue Experiment

The pEX-2 vector (Genepharma, China) was also used for construction of overexpression plasmid of HIF-1α and β-catenin. The co-transfection of constructed plasmid was performed in each cell line using Lipofectamine™ 3000 Transfection Reagent (Invitrogen, USA). Concretely, the co-transfection was classified as the following groups: (a) β-catenin(+)&AKIP1(−), the cells were transfected with β-catenin overexpression plasmid and AKIP1(−) plasmid; (b) β-catenin(+), the cells were transfected with β-catenin overexpression plasmid and NC(−) plasmid; (c) HIF-1α(+)&AKIP1(−), the cells were transfected with HIF-1α overexpression plasmid and AKIP1(−) plasmid; (d) HIF-1α(+), the cells were transfected with HIF-1α overexpression plasmid and NC(−) plasmid; (e) AKIP1(−), the cells were transfected with AKIP1(−) plasmid; (f) NC, the cells were transfected with NC(+) and NC(−) plasmid; and (g) normal, the cells were not transfected with any plasmid. After transfection, all cells were incubated for 48 h under the hypoxia condition as described in the “*Cell Culture and Hypoxia Treatment*”. The Transwell assay, flowcytometry, sphere formation assay, RT-qPCR, and Western blot were performed.

### Transwell Assay

The cells (1 × 10^5^ cells) in 100 μl medium without fetal bovine serum (FBS) were seeded into Matrigel matrix (1.5 mg/ml)-coated insert (Invitrogen, USA). The lower chamber was filled with 10% FBS-containing medium. After incubation for another 24 h, the non-invading cells were removed from the upper surface of the insert. The cells attached to the bottom surface of insert were fixed with methanol (Sangon, China) and stained with 0.5% crystal violet (Sangon, China). The numbers of invaded cells were counted under an inverted light microscope (Nikon, Japan).

### Flow Cytometry

The CD133-positive (CD133^+^) cells proportion was assessed by flow cytometry. The cells were stained with Alexa Fluor^®^ 488-conjugated CD133 mouse monoclonal antibody (1:50, Invitrogen, USA); following that, the cells were sorted by a flow cytometer (BD, USA) and quantified with FlowJo 7.0 software (BD, USA).

### Sphere Formation Assay

In brief, the single cells (300 cells per well) were seeded in ultra-low attach plate (Corning, China) and cultured with sphere formation medium for 7 days under hypoxia condition. The sphere formation medium was Dulbecco’s modified Eagle’s medium/F12 (GIBCO, USA) containing 2% B-27 supplement (Gibco, USA), 20 ng/ml epidermal growth factor (Sangon, China), 10 ng/ml basic fibroblast growth factor (Sangon, China), and 5 μg/ml insulin (Sangon, China). The number of spheres, with diameter >50 μm, was counted with an inverted microscope.

### RT-qPCR

Briefly, total RNA was extracted from cells using TRIzol™ Reagent (Thermo, USA), and the reverse transcription was completed using ReverTra Ace^®^ qPCR RT Kit (Toyobo, Japan); then, qPCR was conducted using SYBR^®^ Green Real-Time PCR Master Mix (Toyobo, Japan). β-Actin was used as reference gene. The primers were as follows: AKIP1 forward primer (5′–3′), AGAACATCTCTAAGGACCTCTACAT; reverse primer (5′–3′), TCCAGAATCAACTGCTACCACAT; HIF-1α forward primer (5′–3′), AACTAGCCGAGGAAG AACTATGAAC; reverse primer (5′–3′), CACTGAGGTTGGT TACTGTTGGT; β-catenin forward primer (5′–3′), GCCATTACAACTCTCCACAACCT; reverse primer (5′–3′), GACAGATAGCACCTTCAGCACTC; β-actin, forward primer (5′–3′), TCGTGCGTGACATTAAGGAGAA; reverse primer (5′–3′), AGGAAGGAAGGCTGGAAGAGT. The expression was finally calculated using 2^−ΔΔCt^ method with β-actin as internal reference.

### Western Blot

Protein extraction and quantification were completed using radioimmunoprecipitation assay (RIPA) lysis and extraction buffer (Thermo, USA) and Pierce™ BCA Protein Assay Kit (Thermo, USA), respectively. Protein was separated by gel electrophoresis with NuPAGE Bis–Tris gels 4%–20% (Thermo, USA), then was transferred onto polyvinylidene difluoride membranes. Subsequently, the membrane was blocked and incubated with antibodies targeting AKIP1 (1:1,000, Invitrogen, USA), HIF-1α (1:1,000, Abcam, UK), vascular endothelial growth factor (VEGF) (1:10,000, Abcam, UK), β-catenin (1:2,000, Invitrogen, USA), calcium-binding protein (CBP) (1:2,000, Invitrogen, USA), IκBα (1:3,000, Invitrogen, USA), p-IκBα (1:1,000, Invitrogen, USA), and β-actin (1:2,000, Invitrogen, USA) (as reference), followed by incubation with goat antirabbit IgG H&L (HRP) secondary antibody (1: 10,000, Abcam, UK). The protein band visualization was performed using Novex™ECL Chemiluminescent Substrate Reagent Kit (Invitrogen, USA). Quantitative analysis was completed using ImageJ software 7.6 (National Institutes of Health, USA).

### Statistical Analysis

Figures were constructed by GraphPad Prism 7.01 (GraphPad, USA) and were displayed by a bar plot using mean and standard deviation. Independent-samples *t*-test was applied for the analysis of the data between two groups, and one-way ANOVA followed by Tukey’s multiple comparison was applied to compare the difference among groups. A *p*-value <0.05 was used as threshold to identify the statistical significance. In the figures, “*,” “**,” “***,” and “NS” represented the *p-*values <0.05, <0.01, <0.001, and >0.05, respectively.

## Results

### AKIP1, Cell Invasion, and Stemness in Gastric Cancer Under Hypoxia

**Figure 1:**
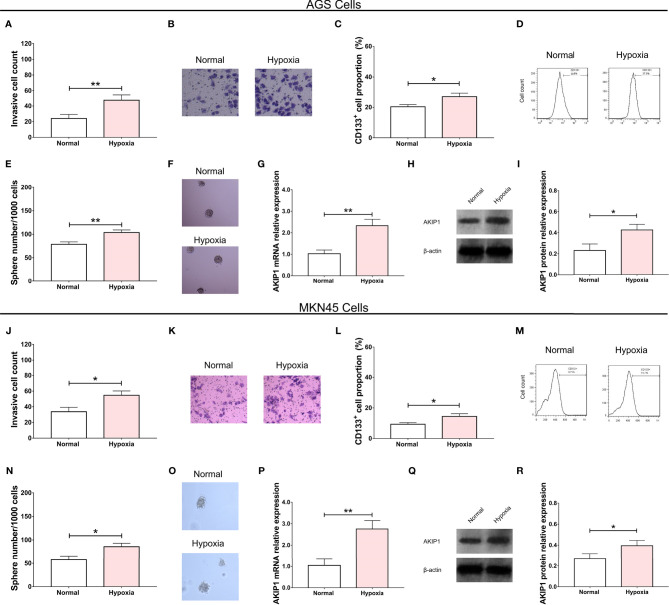
Effect of hypoxia condition on gastric cancer cells. The effect of hypoxia condition on invasive cell number **(A, B)**, CD133^+^ cell proportion **(C, D)**, sphere number/1,000 cells **(E, F)**, and AKIP1 expression **(G–I)** in AGS cells; effect of hypoxia condition on invasive cell number **(J, K)**, CD133^+^ cell proportion **(L, M)**, sphere number/1,000 cells **(N, O)**, and AKIP1 expression **(P, Q, R)** in MKN45 cells. AKIP1, A-kinase interacting protein 1. “*” and “**” represented the p-values <0.05 and <0.01, respectively.

In the AGS cells, the invasive cell count (*p* < 0.01, **Figures 1A, B**), CD133^+^ cell proportion (*p* < 0.05, **Figures 1C, D**), and sphere number/1,000 cells (*p* < 0.01, **Figures 1E, F**) were all elevated in the hypoxia group compared with that in the normal group. The AKIP1 mRNA relative expression (*p* < 0.01, **Figure 1G**) and protein expression (*p* < 0.05, **Figures 1H, I**) were also increased in the hypoxia group compared with that in the normal group. Similar trends were also observed in MKN45 cells (all *p* < 0.05, **Figures 1J–R**). These suggested that AKIP1 might be engaged in the hypoxia-induced cell invasion and stemness in gastric cancer.

### Effect of AKIP1 on Gastric Cancer Cell Invasion and Stemness Under Hypoxia

**Figure 2:**
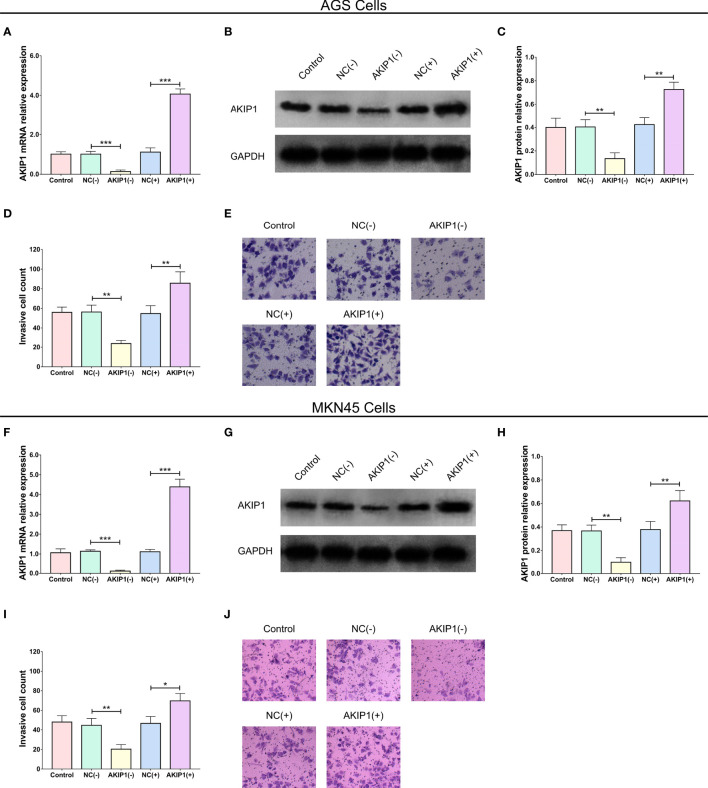
AKIP1 expression and cell invasion in gastric cancer cells post transfections. The AKIP1 mRNA expression **(A)**, protein expression **(B, C)**, and invasive cell count **(D, E)** among control, NC(−), AKIP1(−), NC(+), and AKIP1(+) groups in AGS cells; AKIP1 mRNA expression **(F)**, protein expression **(G, H)**, and invasive cell count **(I, J)** among control, NC(−), AKIP1(−), NC(+), and AKIP1(+) groups in MKN45 cells. AKIP1, A-kinase interacting protein 1; NC, negative control; GAPDH, glyceraldehyde-phosphate dehydrogenase. “*”, “**”, and “***” represented the p-values <0.05, <0.01, and <0.001 respectively.

After the transfection of AKIP1(+) and AKIP1(−) plasmids in AGS cells, AKIP1 mRNA (*p* < 0.001), and protein expressions (*p* < 0.01) were downregulated in the AKIP1(−) group compared with that in the NC(−) group, while they were upregulated in AKIP1(+) compared with that in NC(+) group (both *p* < 0.01, **Figures 2A–C**). Besides, similar trends were also observed in MKN45 cells (all *p* < 0.01, **Figures 2F–H**). These findings suggested the successful transfection of AKIP1(+) and AKIP1(−) plasmids.

More importantly, the invasive cell count was reduced in the AKIP1(−) group compared with that in the NC(−) group (*p* < 0.01) but was enhanced in the AKIP1(+) group compared with that in the NC(+) group (*p* < 0.01, **Figures 2D, E**) after transfections in AGS cells, and similar results were seen in MKN45 cells as well (both *p* < 0.05, **Figures 2I, J**).

**Figure 3:**
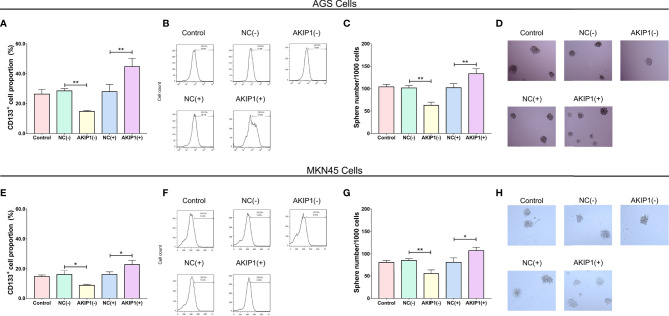
Stemness in gastric cancer cells post transfections. The CD133^+^ cell proportion **(A, B)** and sphere number/1,000 cells **(C, D)** among control, NC(−), AKIP1(−), NC(+), and AKIP1(+) groups in AGS cells; CD133^+^ cell proportion **(E, F)** and sphere number/1,000 cells **(G, H)** among control, NC(−), AKIP1(−), NC(+), and AKIP1(+) groups in MKN45 cells. NC, negative control; AKIP1, A-kinase interacting protein 1. “*” and “**” represented the p-values <0.05 and <0.01, respectively.

In addition, the CD133^+^ cell proportion (*p* < 0.01, **Figures 3A, B**) and sphere number/1,000 cells (*p* < 0.01, **Figures 3C, D**) were downregulated in the AKIP1(−) group compared with that in the NC(−) group but were upregulated in the AKIP1(+) group compared with that in the NC(+) group in AGS cells (both *p* < 0.01). Besides, similar trends were also observed in MKN45 cells (all *p* < 0.05, **Figures 3E–H**). These data revealed that AKIP1 could promote cell invasion and stemness of gastric cancer cells under hypoxia.

### Effects of AKIP1 on HIF-1α and β-Catenin Pathways

**Figure 4:**
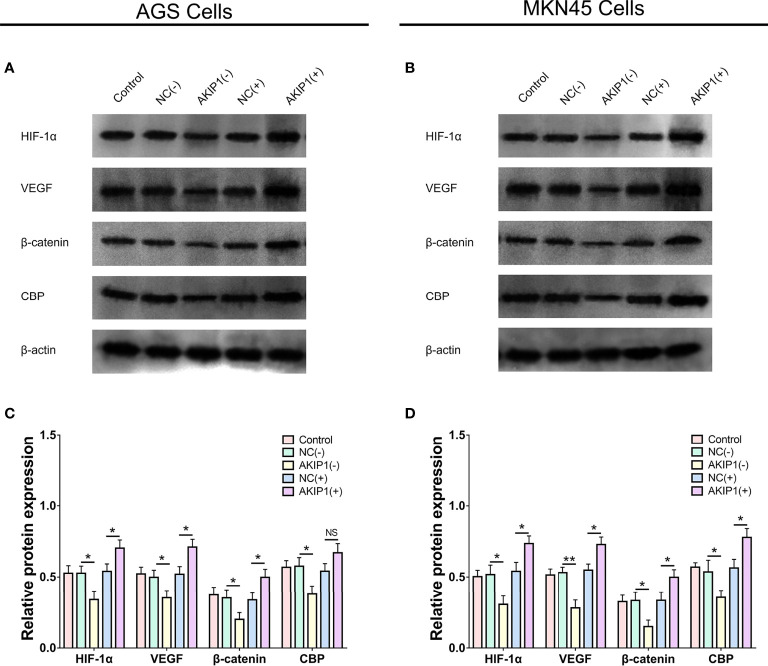
HIF-1α and β-catenin pathways in gastric cancer cells post transfections. The HIF-1α, VEGF, β-catenin, and CBP protein expressions among NC(−), AKIP1(−), NC(+), and AKIP1(+) groups in AGS cells **(A, C)**; HIF-1α, VEGF, β-catenin, and CBP protein expressions among NC(−), AKIP1(−), NC(+), and AKIP1(+) groups in MKN45 cells **(B, D)**. HIF-1α, hypoxia inducible factor 1 subunit alpha; VEGF, vascular endothelial growth factor; CBP, calcium-binding protein; NC, negative control; AKIP1, A-kinase interacting protein 1. “ns” represented the p-value > 0.05. “*” and “**” represented the p-values <0.05 and <0.01, respectively.

In AGS cells, HIF-1α (*p* < 0.05), VEGF (*p* < 0.05), β-catenin (*p* < 0.05), and CBP (*p* < 0.05) protein expressions were inhibited in the AKIP1(−) group compared with that in the NC(−) group, while most of them were promoted in the AKIP1(+) group compared with that in the NC(+) group (*p* < 0.05) except that the CBP expression was of no difference between the AKIP1(+) group compared with that in the NC(+) group (*p* > 0.05) (**Figures 4A, C**).

In the MKN45 cells, the HIF-1α, VEGF, β-catenin, and CBP protein expressions were also decreased in the AKIP1(−) group compared with that in the NC(−) group (all *p* < 0.05) but were increased in the AKIP1(+) group compared ith the NC(+) group (all *p* < 0.05, **Figures 4B, D**). To sum up, these indicated that AKIP1 could positively regulate HIF-1α and β-catenin pathways in gastric cancer cells under hypoxia.

To further evaluate the pharmacological inhibition of HIF1 and beta-catenin in both AGS and MKN45 cell lines, we purchased YC-1 and LF3, the inhibitors of HIF-1α and β-catenin pathways, and treated AGS and MKN45 cell lines with these agents. It showed that the pharmacological inhibition of HIF1 and beta-catenin had the similar effect as AKIP1 depletion on invasive cell count, sphere number/1,000 cells, and CD133+ cell proportion (all *p* < 0.05, **Supplementary Figures 1A–L**).

### Effects of AKIP1 on IκBα and p-IκBα

In AGS cells, p-IκBα protein expression was inhibited in the AKIP1(−) group compared with that in the NC (−) group (*p* < 0.01), while it was promoted in the AKIP1(+) group compared with that in the NC(+) group (*p* < 0.001) (**Supplementary Figures 2A, C**). In the MKN45 cells, p-IκBα protein expression was also inhibited in the AKIP1(−) group compared with that in the NC(−) group (*p* < 0.001), while it was promoted in the AKIP1(+) group compared with that in the NC(+) group (*p* < 0.001) (**Supplementary Figures 2B, D**). To sum up, these indicated that AKIP1 could positively regulate p-IκBα expression in gastric cancer cells under hypoxia.

### Rescue Experiments for Interaction of AKIP1 With HIF-1α and β-Catenin Pathways

**Figure 5:**
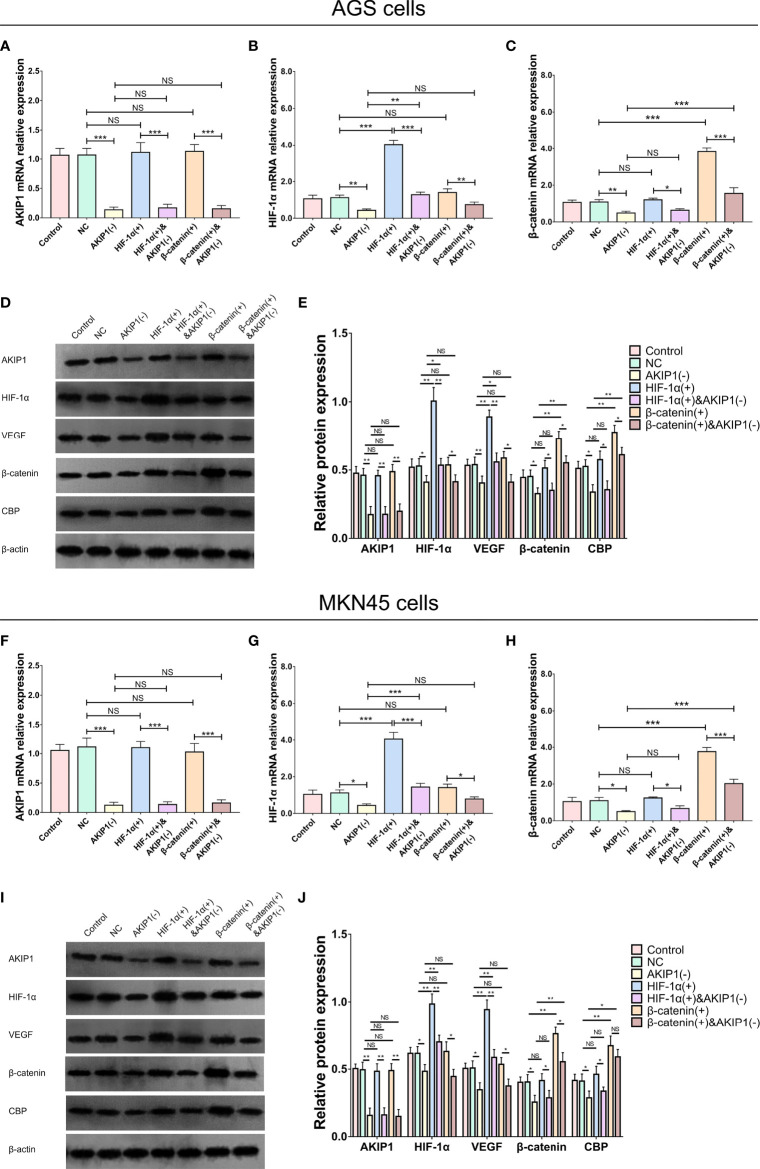
AKIP1, HIF-1α, and β-catenin expressions in rescue experiments. The AKIP1 mRNA **(A)**, HIF-1α mRNA **(B)**, β-catenin mRNA **(C)**, and their protein **(D, E)** expressions among control, NC, AKIP1(−), HIF-1α(+), HIF-1α(+)&AKIP1(−), β-catenin(+), and β-catenin(+)&AKIP1(−) groups in AGS cells; AKIP1 mRNA **(F)**, HIF-1α mRNA **(G)**, β-catenin mRNA **(H)**, their protein **(I, J)** expressions among control, NC, AKIP1(−), HIF-1α(+), HIF-1α(+)&AKIP1(−), β-catenin(+), and β-catenin(+)&AKIP1(−) groups in MKN45 cells. AKIP1, A-kinase interacting protein 1; HIF-1α, hypoxia-inducible factor 1 subunit alpha; NC, negative control; VEGF, vascular endothelial growth factor; CBP, calcium-binding protein; NS, not significant. “*”, “**”, and “***” represented the p-values <0.05, <0.01, and <0.001 respectively.

In AGS cells, the AKIP1 mRNA (*p* < 0.001) and protein expressions (*p* < 0.01) were downregulated in the AKIP1(−) group compared with that in the NC group, which indicated that the transfection of AKIP1 knockdown plasmid was successful; the HIF-1α mRNA (*p* < 0.001) and protein expressions (*p* < 0.01) were overexpressed in the HIF-1α(+) group compared with that in the NC group, which indicated that the successful transfection of HIF-1α overexpression plasmid; the AKIP1 mRNA (*p* < 0.001) and protein expressions (*p* < 0.01) were downregulated in the HIF-1α&(+)&AKIP1(−) group compared with that in the HIF-1α(+) group, which indicated that the transfection of HIF-1α overexpression and AKIP1 knockdown plasmid was successful; the β-catenin mRNA (*p* < 0.001) and protein expressions (*p* < 0.05) were overexpressed in the β-catenin(+) group compared with that in the NC group, which indicated that the successful transfection of β-catenin overexpression plasmid; the AKIP1 mRNA (*p* < 0.001) and protein expressions (*p* < 0.01) were downregulated in the β-catenin(+)&AKIP1(−) group compared with that in the β-catenin(+) group, which indicated that the transfection of β-catenin overexpression and AKIP1 knockdown plasmid was successful (**Figures 5A–E**). In MKN45 cells, similar results were also observed, which indicated that the transfections were also successful (**Figures 5F–J**).

**Figure 6:**
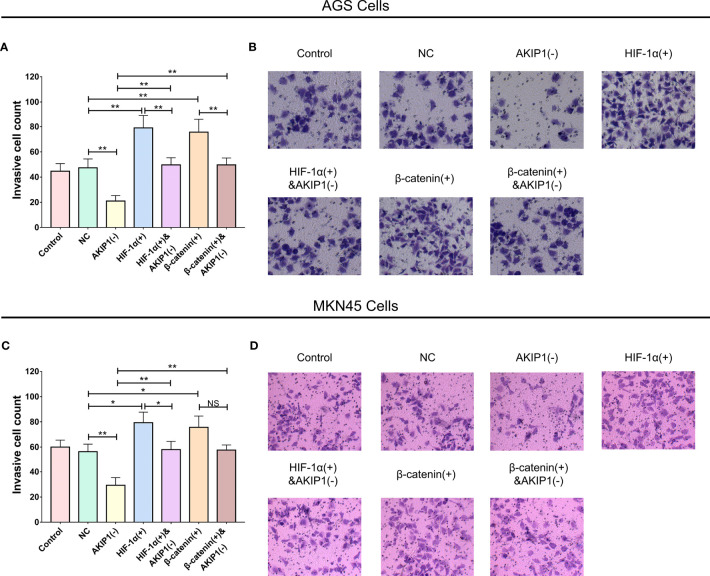
Cell invasion in rescue experiments. The invasive cell number among control, NC, AKIP1(−), HIF-1α(+), HIF-1α(+)&AKIP1(−), β-catenin(+), and β-catenin(+)&AKIP1(−) groups in AGS cells **(A, B)** and in MKN45 cells **(C, D)**. AKIP1, A-kinase interacting protein 1; NC, negative control; HIF-1α, hypoxia-inducible factor 1 subunit alpha; NS, not significant. “*” and “**” represented the p-values <0.05 and <0.01, respectively.

Furthermore, in AGS cells, the invasive cell count was elevated in the HIF-1α(+) group compared with thar in the NC group (*p* < 0.01); besides, the invasive cell count was also elevated in the HIF-1α(+)&AKIP1(−) group compared with that in the AKIP1(−) group (*p* < 0.01), but it was still declined in the HIF-1α(+)&AKIP1(−) group compared with that in the HIF-1α(+) group (*p* < 0.01). These findings indicated that HIF-1α overexpression elevated invasive cell count, and HIF-1α overexpression might diminish the effect of AKIP1 knockdown on invasive cell count to some extent; in addition, the invasive cell count was elevated in the β-catenin(+) group compared with that in the NC group (*p* < 0.01), besides the invasive cell count was also elevated in the β-catenin(+)&AKIP1(−) group compared with that in the AKIP1(−) group (*p* < 0.01), but it was still declined in the β-catenin(+)&AKIP1(−) group compared with that in the β-catenin(+) group (*p* < 0.01). These finding indicated that β-catenin overexpression elevated invasive cell count, and β-catenin overexpression might decrease the effect of AKIP1 knockdown on invasive cell count to some extent (**Figures 6A, B**). In MKN45 cells, HIF-1α and β-catenin overexpressions exhibited a similar impact on invasive cell count, and they both weaken the effect of AKIP1 knockdown on invasive cell count to some extent (**Figures 6C, D**).

**Figure 7:**
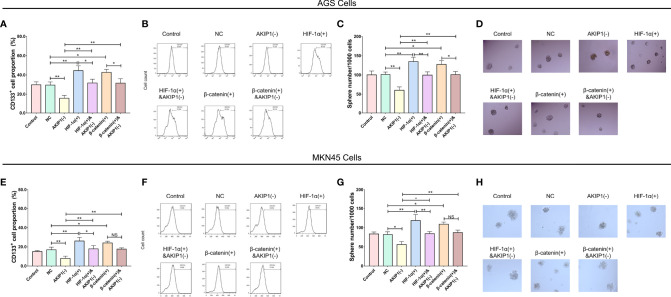
Cell stemness in rescue experiments. CD133^+^ cell proportion and sphere number/1,000 cells among control, NC, AKIP1(−), HIF-1α(+), HIF-1α(+)&AKIP1(−), β-catenin(+), and β-catenin(+)&AKIP1(−) groups in AGS cells **(A–D)** and in MKN45 cells **(E–H)**, respectively. NC, negative control; AKIP, A-kinase interacting protein 1; HIF-1α, hypoxia-inducible factor 1 subunit alpha; NS, not significant. “*” and “**” represented the p-values <0.05 and <0.01, respectively.

In addition, in AGS cells, the CD133^+^ cell proportion (*p* < 0.01) and sphere number/1,000 cells (*p* < 0.01) were elevated in the HIF-1α(+) group compared with that in the NC group (*p* < 0.01); besides, the CD133^+^ cell proportion (*p* < 0.01) and sphere number/1,000 cells (*p* < 0.01) were also elevated in the HIF-1α(+)&AKIP1(−) group compared with that in the AKIP1(−) group (*p* < 0.01), but they were still declined in the HIF-1α(+)&AKIP1(−) group compared with that in the HIF-1α(+) group (both *p* < 0.05). These findings indicated that HIF-1α overexpression elevated the CD133^+^ cell proportion and sphere number/1,000 cells, and HIF-1α overexpression might reduce the effect of AKIP1 knockdown on these measurements to some extent. In addition, the CD133^+^ cell proportion (*p* < 0.01) and sphere number/1,000 cells (*p* < 0.01) were elevated in the β-catenin(+) group compared with that in the NC group (*p* < 0.01); besides, the CD133^+^ cell proportion (*p* < 0.01) and sphere number/1,000 cells (*p* < 0.01) were also elevated in the β-catenin(+)&AKIP1(−) group compared with that in the AKIP1(−) group (*p* < 0.01), but they were still declined in the β-catenin(+)&AKIP1(−) group compared with that in the β-catenin(+) group (both *p* < 0.05). These findings indicated that β-catenin overexpression elevated the CD133^+^ cell proportion and sphere number/1,000 cells, and β-catenin overexpression might curtail the effect of AKIP1 knockdown on these measurements to some extent (**Figures 7A–D**). In MKN45 cells, HIF-1α and β-catenin overexpressions exhibited a similar impact on CD133^+^ cell proportion and sphere number/1,000 cells, and they both attenuated the effect of AKIP1 knockdown on these measurements to some extent (**Figures 7E–H**).

## Discussion

Caused by cancer cell overproliferation, oxygen deficiency is a common phenomenon inside the tumor; most importantly, this phenomenon is increasingly reported as a potential treatment target in cancers (21, 22). The hypoxia condition is reported to promote tumor progression in multiple ways, such as the enhancement of chemoresistance by activating GLI family zinc finger 2 (GLI2) and promotion of tumor metastasis through modulating the macrophage functions (23, 24). As for gastric cancer, there are also studies reporting the role of hypoxia condition. For instance, a study elucidated that hypoxia promotes gastric cancer cell migration, invasion, and epithelial–mesenchymal transition (EMT); then, circular RNA 0081143 knockdown reduces these effects (25). Another study revealed that the axis of hypoxia–autophagy enhances the secretion of vascular endothelial growth factor A (VEGFA) from peritoneal mesothelial cells through mediating integrin α5-fibronectin signaling, which subsequently promotes the peritoneal metastasis in gastric cancer mouse model (26). In addition, a study illuminated that microRNA 124 expression is activated by hypoxia, which then increases cancer cell proliferation and migration by upregulating the Warburg effect (27). In the present study, we tried to explore the role of AKIP1 in gastric cancer cells under hypoxia and its probable downstream pathways and found that, first of all, the cell invasion, CD133^+^ cell percentage, and sphere number/1,000 cells were promoted in gastric cancer cells under hypoxia. AKIP1 was upregulated in gastric cancer cells under hypoxia, and AKIP1 enhanced cell invasion, CD133^+^ cell proportion, and sphere number/1,000 cells in gastric cancer cells under hypoxia. Furthermore, the rescue experiments disclosed that HIF-1α and β-catenin pathways overexpression reduced the effects of AKIP1 knockdown on invasion, CD133^+^ cell proportion, and sphere number/1,000 cells in gastric cancer cells under hypoxia.

AKIP1, also known as breast-cancer-associated protein 3 (BCA3), was earlier found as a regulator of cAMP-dependent protein kinase (PKA) that modulates the cardiac function, and now it is also known as a factor related to oncology (14). A previous study illustrates that AKIP1 promotes the malignant behaviors of hepatocellular carcinoma (HCC) cells *via* activating protein kinase B (AKT) and nuclear factor-kappa B (NF-kB) translocation (28). Moreover, a study revealed that in esophageal squamous cell carcinoma, *in vivo* and *in vitro*, AKIP1 overexpression enhances angiogenesis and lymphangiogenesis, and AKIP1 downregulation exerts the opposite effects. The study also reported that AKIP1 enhances VEGF-C through regulating its promotor by interacting with the transcription factors consisting of SP1, AP2, and NF-kB (29). Besides, AKIP1 inhibition results in reduction in tumor growth and angiogenesis in cervical cancer mouse models and decrease in proliferation in cervical cancer cells (30). These all indicate that AKIP1 acts as a promotor in the development of cancers, which is in consistence to our results. Our results elucidated that AKIP1 promoted migration and stemness of gastric cancer cells, suggesting that it also acted as a promoter of tumor progression in gastric cancer. In regard to the possible regulatory mechanism of AKIP1 in gastric, it possibly promoted gastric cancer cell invasion and stemness through interacting with other oncology-related factors (such as AKT and NF-kB), or as elucidated in our further experiments, AKIP1 could also regulate the gastric cancer cell functions *via* upregulating HIF-1α and β-catenin pathways (14, 28–30).

As for the roles of HIF-1α and β-catenin pathways, they are both involved in tumorigenesis, which includes the pathology of gastric cancer. For example, the gastric cancer cell proliferation and invasion could be inhibited by 3-deazaneplanocin A through repressing of the HIF-1α and Wnt signaling pathways (31). In addition, a study elucidates that the molecule collagen triple helix repeat containing 1 (CTHRC1) enhances the tumor metastasis in animal model of gastric cancer *via* positively mediating HIF-1α expression (32). In addition, a study illuminates that the Wnt/β-catenin signaling pathway promotes the metastasis and EMT-related markers expressions *via* interacting with transcription factor EB (TFEB) in gastric cancer cells (33). Besides, a study revealed that the Wnt/β-catenin signaling pathway is essential in the immunosuppression regulation in gastric cancer microenvironment, and inhibiting the β-catenin-induced C–C motif chemokine ligand 28 (CCL28) decreases the tumor progression through downregulating Treg cell infiltration in animal models (34). These findings indicate that these two pathways might be critical in gastric cancer progression. In the present study, we discovered that AKIP1 positively regulated the HIF-1α and β-catenin pathways in gastric cancer cells under hypoxia, and both HIF-1α and β-catenin overexpressions could diminish the effects of AKIP1 on gastric cancer cell invasion and stemness under hypoxia, which has provided some more possible explanations to the function of AKIP1 in gastric cancer.

To sum up, AKIP1 promotes cell invasion and stemness *via* activating HIF-1α and β-catenin pathways in gastric cancer under hypoxia, which indicates that AKIP1 may have the potential to serve as a therapeutic target in gastric cancer.

## Data Availability Statement

The raw data supporting the conclusions of this article will be made available by the authors, without undue reservation.

## Author Contributions

ZL and YL contributed to the study design and manuscript writing. ZL, YL, and KX conducted literature research and cultured GC cells. ZL and YL contributed to the data acquisition and analysis. KX critically revised the manuscript for important intellectual content. All authors contributed to the article and approved the submitted version. All authors agree to be accountable for all aspects of the work in ensuring that questions related to the accuracy or integrity of the work are appropriately investigated and resolved.

## Funding

This study was supported by the General guiding project of Hunan Provincial Health Commission(no. 202204014314).

## Conflict of Interest

The authors declare that the research was conducted in the absence of any commercial or financial relationships that could be construed as a potential conflict of interest.

## Publisher’s Note

All claims expressed in this article are solely those of the authors and do not necessarily represent those of their affiliated organizations, or those of the publisher, the editors and the reviewers. Any product that may be evaluated in this article, or claim that may be made by its manufacturer, is not guaranteed or endorsed by the publisher.

## Supplementary Material

Supplementary Figure 1 Pharmacological inhibition of HIF1 and beta-catenin on cell invasion and stemness in gastric cancer cells. Invasive cell count, CD133^+^ cell proportion, and sphere number/1000 cells among control, NC, AKIP1(-), HIF-1α(-), and β-catenin(-) groups in AGS cells **(A–F)** and in MKN45 cells **(G–L)**, respectively. NC, negative control; AKIP, A-kinase interacting protein 1; HIF-1α, hypoxia inducible factor 1 subunit alpha; NS, not significant.

Supplementary Figure 2 IκBα and p-IκBα in gastric cancer cells post transfections. The IκBα and p-IκBα protein expressions among control, NC(-), AKIP1(-), NC(+) and AKIP1(+) groups in AGS cells **(A, C)**; The IκBα and p-IκBα protein expressions among control, NC(-), AKIP1(-), NC(+) and AKIP1(+) groups in MKN45 cells **(B, D)**. IκBα, nuclear factor-κB inhibitory protein-α; p-IκBα, phosphorylated-IκBα; NC, negative control; AKIP1, A-kinase interacting protein 1.
