# Supplementary figures and images for: A-Kinase Interacting Protein 1 Promotes Cell Invasion and Stemness via Activating HIF-1α and β-Catenin Signaling Pathways in Gastric Cancer Under Hypoxia Condition
Source: Front Oncol. 2022 Mar 9;11:798557. doi: 10.3389/fonc.2021.798557 (PMC8959465; doi:10.3389/fonc.2021.798557)

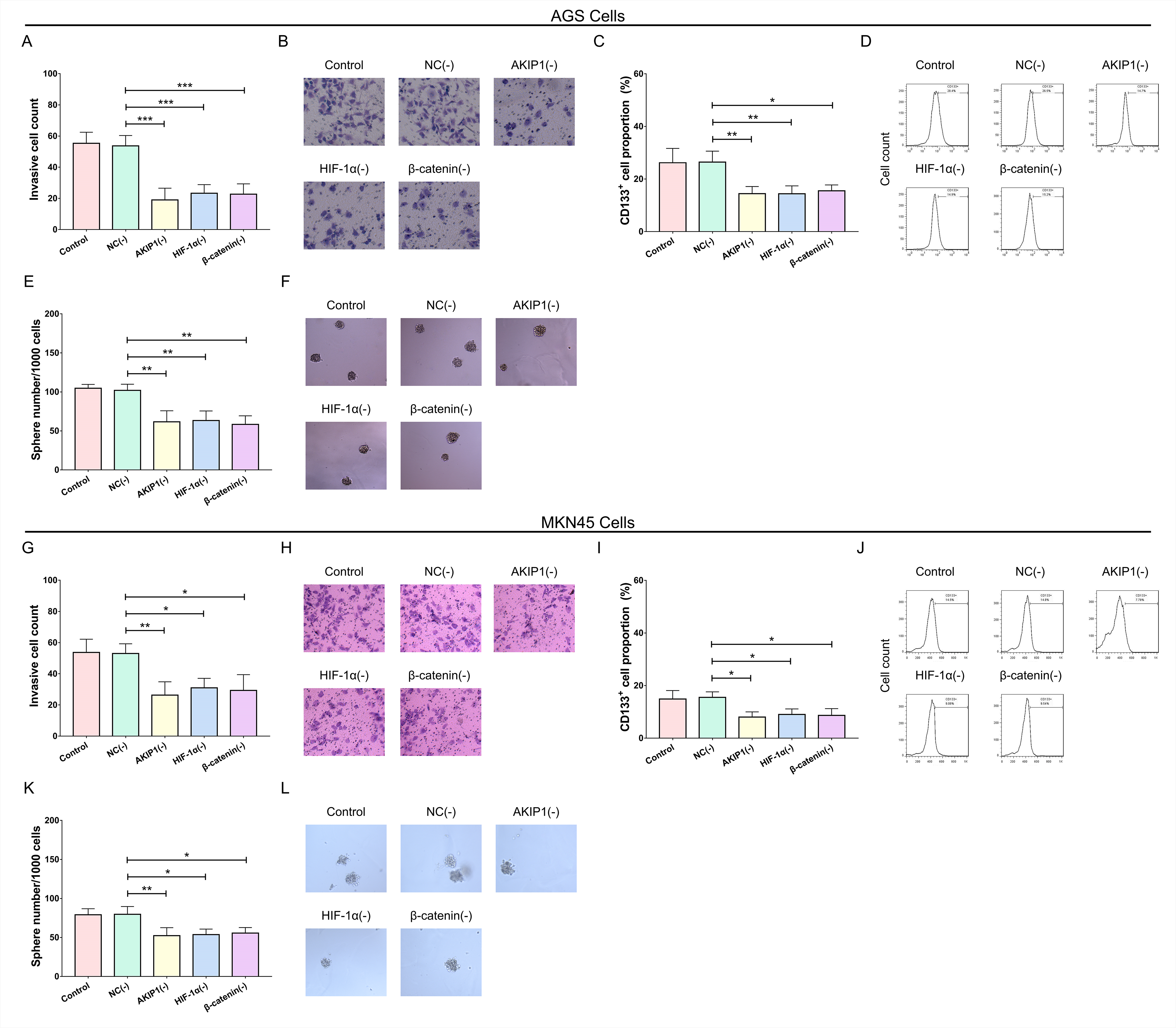

Supplement: Supplementary Figure 1 — Pharmacological inhibition of HIF1 and beta-catenin on cell invasion and stemness in gastric cancer cells. Invasive cell count, CD133+ cell proportion, and sphere number/1000 cells among control, NC, AKIP1(-), HIF-1α(-), and β-catenin(-) groups in AGS cells (A–F) and in MKN45 cells (G–L), respectively. NC, negative control; AKIP, A-kinase interacting protein 1; HIF-1α, hypoxia inducible factor 1 subunit alpha; NS, not significant. [file Image_1.tif]

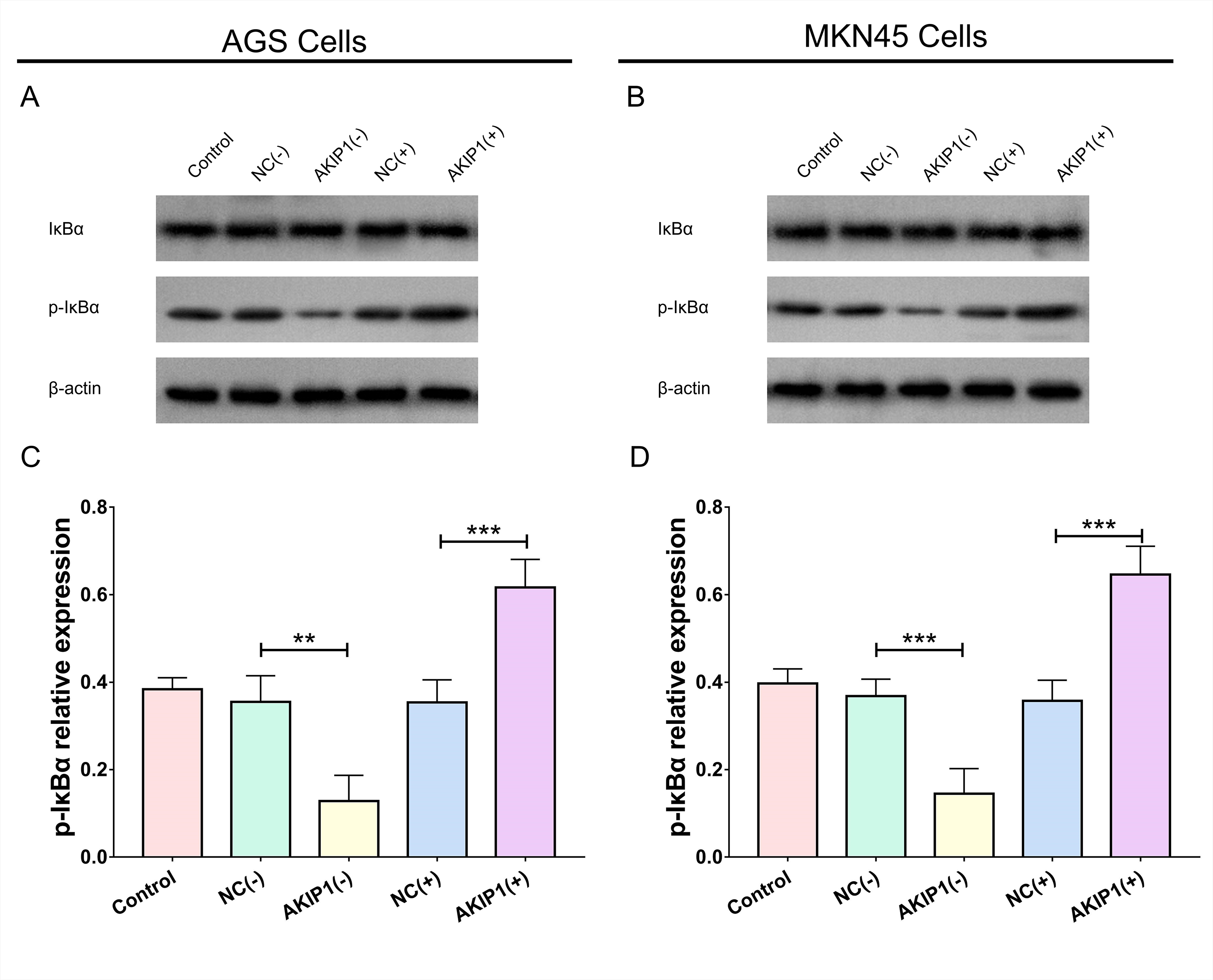

Supplement: Supplementary Figure 2 — IκBα and p-IκBα in gastric cancer cells post transfections. The IκBα and p-IκBα protein expressions among control, NC(-), AKIP1(-), NC(+) and AKIP1(+) groups in AGS cells (A, C); The IκBα and p-IκBα protein expressions among control, NC(-), AKIP1(-), NC(+) and AKIP1(+) groups in MKN45 cells (B, D). IκBα, nuclear factor-κB inhibitory protein-α; p-IκBα, phosphorylated-IκBα; NC, negative control; AKIP1, A-kinase interacting protein 1. [file Image_2.tif]
